# Influence of PGPB Inoculation on *HSP70* and *HMA3* Gene Expression in Switchgrass under Cadmium Stress

**DOI:** 10.3390/plants8110504

**Published:** 2019-11-14

**Authors:** Nahmina Begum, Zhaoyang Hu, Qingsheng Cai, Laiqing Lou

**Affiliations:** College of Life Sciences, Nanjing Agricultural University, Nanjing 210095, China; 2016216002@njau.edu.cn

**Keywords:** agriculture, Cd stress, environmental, gene expression, PGPB, switchgrass

## Abstract

This study aimed to evaluate the gene expression of *HSP70* and *HMA3* in the switchgrass inoculated with plant-growth-promoting-bacteria (PGPB) under cadmium (Cd) stress and to observe the benefit of PGPB in plant growth and development. Plants were grown in hydroponic culture and treated with PGPB inoculants: *Pseudomonas grimontii*, *Pantoea vagans*, *Pseudomonas veronii*, and *Pseudomonas fluorescens* with the strains Bc09, So23, E02, and Oj24, respectively. The experimental results revealed that *HSP70* and *HMA3* genes expressed highly in the PGPB-inoculated plants under Cd stress. In addition, the expression of *HSP70* and *HMA3* genes was considerably higher in the first two days after successive four-day exposure of Cd in plants compared to the last two days of exposure. Increased biomass and indole-3-acetic-acid production with reduced Cd accumulation were observed in the PGPB-inoculated plants under Cd stress compared to the Cd-control plants. These PGPB, with their beneficial mechanisms, protect plants by modifying the gene expression profile that arises during Cd-toxic conditions and increased the healthy biomass of switchgrass. This demonstrates there is a correlation among the growth parameters under Cd stress. The PGPB in this study may help to intensify agriculture by triggering mechanisms to encourage plant growth and development under heavy metal stress.

## 1. Introduction

Currently, biotic and abiotic stresses frequently limit plant production. Heavy metal pollution is a topic of great interest among all abiotic stresses [1,2]. Heavy metal pollution endangers plants, animals, and humans and it is now an alarming global problem. Heavy metal exposure has been shown to cause changes in plant protein expression [1]. In particular, cadmium (Cd) is a threat to living organisms because it accumulates in plants in contaminated environment and can enter the food chain [3]. Cd disturbs normal plant metabolism and results in poor growth and development in host plants [4]. Plants have developed diverse mechanisms for the alleviation of stress and re-establishment of cellular homeostasis, to include expression of metal transporters for metal uptake and vacuolar transport, chelators for tolerance and detoxification of heavy metals, and chaperones that deliver and traffic metal ions [5,6]. Heat-shock proteins (*HSP*s) are well established chaperones involved in “housekeeping” the cell processes [7]. *HSP*s with molecular weight from 10 to 200 kDa participate in cell signaling under stress conditions [8]. The principal *HSP*s are split into five conserved classes according to their approximate molecular weight: *HSP100*, *HSP90*, *HSP70*, *HSP60*, and small heat shock proteins (*sHSPs* of 15 to 42 kDa in molecular weight) [9]. Exposure to Cd resulted in an increased accumulation of stress-related proteins such as *HSPs*, chaperones, foldases, proteases and pathogenesis-related (PR) proteins in the leaf and root tissues of poplar plants, but root tissues exhibited decreased proteins from the primary metabolism pathways [10]. The root tissues of different grass plants of the Yellowstone National Park exhibited higher s*HSPs* compared to shoot tissues under extreme heat exposure [11]. Under metal toxic conditions, toxic metal ions interfere with the cellular protein homeostasis through the folding process and enhance the production of improperly folded proteins followed by endoplasmic reticulum (ER) stress, resulting in decreased cell viability. Under these circumstances, cells initiate a different quality control system to fine-tune the protein homeostasis [8,12]. The *HSP70* family members have been studied extensively in a wide range of plant species, as they accumulate during the environmental stresses [13], primarily localizing to the cytosol, ER, and mitochondria [8]. Numerous studies have shown a correlation between *HSP70* overexpression and heavy metal tolerance, but the cellular function of *HSP70* under stress has not been completely revealed [14]. Together, the *HSP70* chaperones and co-chaperones prevent the accumulation of nascent proteins as aggregates and confirm the appropriate folding of proteins while transferring the proteins to their destination [15]. One of our research groups has highlighted the inevitable role of the HSP network to normalize the protein function under Cd stress in the switchgrass using deep sequencing [16].

*HMA3* is a member of the heavy metal ATPase (HMA) family [3]. *OsHMA3* is a tonoplast-localized transporter for Cd in the roots of rice (*Oryza sativa*) and when it overexpressed, Cd accumulation in grain is reduced [17]. The overexpression of *OsHMA3* causes higher Cd concentration in roots by enhancing the vacuolar sequestration of Cd in the roots. As a consequence, *OsHMA3* can reduce Cd accumulation in grain efficiently and enhance plant tolerance to Cd [3,18]. Notably, overexpression of *OsHMA3* reduced Cd accumulation in grain without affecting Fe and Zn [19].

Plant-growth-promoting-bacteria (PGPB) can enhance plant growth by different direct and/or indirect mechanisms [20]. PGPB of agronomic importance can diminish the detrimental environmental impacts on plants and help to enhance agriculture [21,22]. Switchgrass (*Panicum virgatum* L.) is a model perennial bioenergy crop. It has a high growth rate, good biomass yield, and minimal nutrient requirements, is highly adapted to grow on marginal lands, and has a wide range of habitats [20,23]. This bioenergy crop is largely dependent on natural arbuscular mycorrhizal fungi (AMF), but little is known about the interaction between switchgrass and AMF [24]. In addition, some exotic endophytic PGPB can also colonize switchgrass, for example, *Paraburkholderia phytofirmans* PsJN, *Bacillus*, *Pseudomonas*, *Pantoea*, and *Enterobacter,* etc. [25,26,27]. Recently, the term induced systemic tolerance (IST) has been proposed to describe PGPB-induced physical and chemical changes that occur in host plants due to the tolerance triggered by PGPB-plants interactions during various abiotic stresses [28]. In our previous report, we explained the details of the four PGPB strains, that is, *Pseudomonas grimontii* strain Bc09, *Pantoea vagans* strain So23, *Pseudomonas veronii* strain E02, and *Pseudomonas fluorescens* strain Oj24. These four strains are highly Cd tolerant, produce indole-3-acetic acid (IAA), have 1-aminocyclopropane-1-carboxylic-acid deaminase (ACCD) activities, can solubilize insoluble phosphate, and can colonize inside switchgrass [27]. These PGPB mechanisms are recognized as the IST that mainly evolved during abiotic stresses as described earlier. In the present study, we were eager to evaluate IAA production by these four PGPB in inoculated-switchgrass under Cd stress. IAA production by many PGPB plays a key role in the growth and development of the plants. IAA can induce tissue differentiation, cell division, and elongation, lateral-root formation, etc. [29]. It is reported that a number of bacteria can produce IAA, for example, *Azospirillum brasilense*, *Enterobacter cloacae* UW5, *Pantoea agglomerans*, and *Pseudomonas putida* [30,31,32,33,34].

Currently, we are unaware of any reports regarding IAA production and *HSP70* and *HMA3* gene expression in switchgrass-microbe interactions under Cd stress. Therefore, this experiment focused on *HSP70* and *HMA3* gene expression as well as IAA production and growth promotion due to the switchgrass-PGPB interaction under Cd stress. Additionally, we observed the correlation between these parameters by performing principal component analysis (PCA).

## 2. Results

### 2.1. Biomass of the PGPB-Inoculated Plants with and without Cd Stress

**Figure 1:**
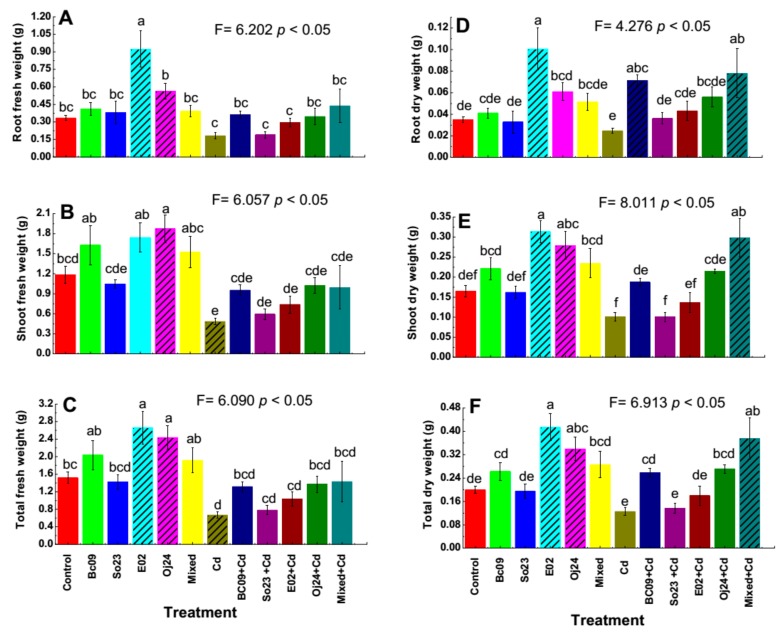
Biomass of switchgrass cv Alamo. (**A**) Root fresh weight, (**B**) shoot fresh weight, (**C**) total fresh weight, (**D**) root dry weight, (**E**) shoot dry weight, and (**F**) total dry weight. Control = noninoculated control plant without Cd stress; Cd = noninoculated Cd-control plant with 20 µ mol L^−1^ CdCl_2_ stress; Bc09, So23, E02, Oj24, and Mixed (combination of the four strain) = plant inoculated with endophytic PGPB without Cd stress; and Bc09 + Cd, So23 + Cd, E02 + Cd, Oj24 + Cd, and Mixed + Cd (combination of the four strain) = plant inoculated with endophytic PGPB with 20 µ mol L^−1^ CdCl_2_ stress. The bar represents ±SE (n = 5). Columns not sharing a significance letter are significantly different according to Duncan’s Multiple Range Test at *p* < 0.05. The dash pattern indicates the biomass of that treatment is significantly different among the treatments.

The plant biomass (fresh and dry weight) of the PGPB-inoculated (single strain and Mixed-combination of the four strain) group under both Cd-amended and Cd-nonamended conditions was measured and is presented in Figure 1A–F. Among the treatments both under Cd stress and without stress, E02-inoculated plants showed significantly higher (*p* < 0.05) root fresh weight (0.9 g) and the next highest root fresh weight (0.6 g) was observed in the Oj24-inoculated plants (Figure 1A). Although the PGPB-inoculated plants with Cd-exposure achieved higher root fresh weight ranging from 3% (3 g in So23 + Cd) to 140% (31 g in Mixed + Cd) compared to the noninoculated Cd-control plants but possessed no significant difference among them (Figure 1A). In the case of root dry weight, among all the treatments E02-inoculated plant (0.1 g) achieved a significantly higher (*p* < 0.05) weight, gradually second and third higher group was found as in Mixed + Cd-inoculated plants (0.08 g) and Bc09 + Cd-inoculated plants (0.07 g) (Figure 1D). The noninoculated Cd-control plants showed lower dry weight (0.02 g) among all the treatments. Under Cd stress, the PGPB-inoculated plants obtained a higher root dry weight than the Cd-control plants that ranged from 46% (0.04 g in So23 + Cd) to 217% (0.08 g in Mixed + Cd) (Figure 1D).

Among all the treatments, Oj24-inoculated plants (1.9 g) exhibited significantly higher (*p* < 0.05) shoot fresh weight and noninoculated Cd-control plants (0.5 g) possessed significantly lower (*p* < 0.05) shoot fresh weight (Figure 1B). Under Cd stress, the PGPB-inoculated plants and noninoculated Cd-control plants exhibited reduced shoot fresh weight compared to the plants that grew normally. However, in the Cd-groups, PGPB-inoculated plants exhibited greater shoot fresh weight, which ranged from 22% (0.6 g in So23 + Cd) to 112% (1 g in Oj24 + Cd) compared to the noninoculated Cd-control plants (Figure 1B), but there was no significant difference among those results. In consideration of shoot dry weight, without Cd stress E02-inoculated (0.3 g) and Oj24-inoculated plants (0.27 g) gained significantly higher (*p* < 0.05) shoot dry weight. Under Cd stress, Mixed + Cd-inoculated plants (0.29 g) exhibited significantly higher (*p* < 0.05) shoot dry weight (Figure 1E). However, all the PGPB-inoculated plants under Cd stress gained from 0.1% (0.1 g in So23 + Cd) to 196% (0.3 g in Mixed + Cd) more shoot dry weight compared to the noninoculated Cd-control plants (Figure 1E). Furthermore, in the Cd-group, Bc09-, E02-, Oj24-, and Mixed-inoculated plants were significantly (*p* < 0.05) different (Figure 1E).

The E02-inoculated plants (2.6 g) and Oj24-inoculated plants (2.4 g) were observed to have significantly higher (*p* < 0.05) total fresh weight, but these two treatments were not significantly different from each other (Figure 1C). A significantly lower (*p* < 0.05) total fresh weight was observed in the noninoculated Cd-control plants (0.7 g) (Figure 1C). Under Cd stress, the plants experienced a reduced total fresh weight compared to under normal conditions. The PGPB-inoculated plants under Cd stress exhibited from 17% (0.8 g in So23 + Cd) to 115% (1 g in Mixed + Cd) more total fresh weight compared to the noninoculated Cd-control plants, although there was no significant difference among them (Figure 1C). The E02-inoculated plants (0.4 g) possessed significantly higher (*p* < 0.05) total dry weight among all the treatments. The next significant higher total dry biomass was observed in the Mixed + Cd-inoculated plants (0.37 g) and Oj24-inoculated plants (0.34 g) (Figure 1F). If we consider only the Cd-group, then PGPB-inoculated plants gained from 9% (0.1 g in So23 + Cd) to 200% (0.4 g in Mixed + Cd) more total dry weight ranged compared to the noninoculated Cd-control plants (Figure 1F). Moreover, plants grown in normal conditions differed significantly with each other, except the control and So23-inoculated plants, while in the Cd-group all the treatments differed significantly with each other except the Cd-control and So23 + Cd treatment.

In this study, all the PGPB-inoculated (single and mixed) and noninoculated plants with and without Cd-exposure were grown under the same environmental conditions at the same time. We observed that Cd-exposure imparts a negative pressure on plants, as the PGPB-inoculated plants under Cd stress showed less biomass in some cases compared to the PGPB-inoculated plants without Cd amendment, but the biomass was greater in the PGPB-inoculated plants under Cd stress compared to the noninoculated Cd-control plant (Figure 1C,F). Among all the treatments, E02-inoculated plants grown without Cd-amended conditions gained significantly (*p* < 0.05) more root and total fresh biomass and root, shoot, and total dry biomass (Figure 1A,C–F). In addition, Oj24-inoculated plants grown without Cd-exposure also achieved significantly more shoot fresh and total biomass (*p* < 0.05) (Figure 1B,C). Furthermore, mixed-inoculated plants grown under Cd stress exhibited significantly (*p* < 0.05) more root, shoot, and total dry biomass (Figure 1D–F). On the other hand, noninoculated Cd-controls possessed significantly (*p* < 0.05) less shoot fresh weight and total fresh weight among all the treatments (Figure 1B,C).

### 2.2. Determination of Cd Concentrations inside Plant Tissues

**Figure 2:**
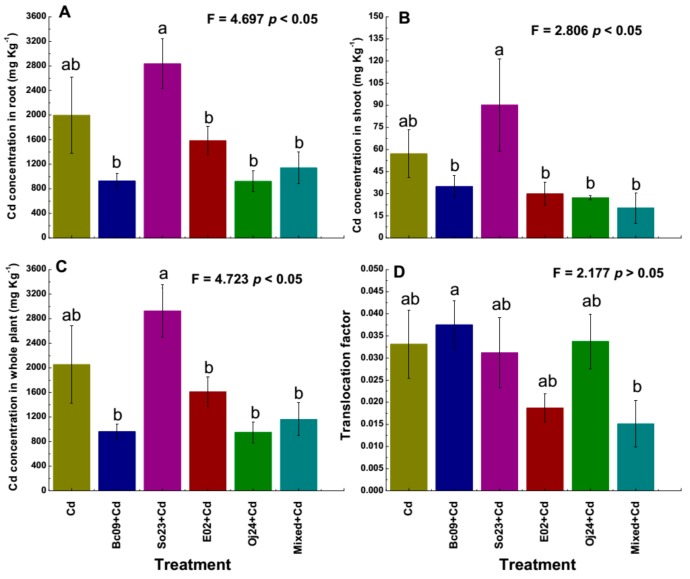
Uptake of Cd in switchgrass cv Alamo under 20 µ mol L^−1^ CdCl_2_ stress. (**A**) Cd concentration in root, (**B**) Cd concentration in shoot, and (**C**) Cd concentration in whole plant, and (**D**) translocation factor (TF). TF represents metal concentration in shoot/root and denotes metal translocation. Cd = noninoculated Cd-control plant with 20 µ mol L^−1^ CdCl_2_ stress and Bc09 + Cd, So23 + Cd, E02 + Cd, Oj24 + Cd, and Mixed + Cd (combination of the four strain) = plant inoculated with endophytic PGPB with 20 µ mol L^−1^ CdCl_2_ stress. The bar represents ± SE (n = 5). Columns not sharing a significance letter are significantly different according to Duncan’s Multiple Range Test at *p* < 0.05.

Cd concentrations in the plant (root, shoot, and whole plant) and the translocation factor (TF) were determined and are presented in Figure 2A–D. The concentrations of Cd in the roots and shoots of the PGPB-inoculated plants were lower compared to those in the noninoculated Cd-control plants, except for the So23-inoculated plants. The So23-inoculated plants exhibited significantly (*p* < 0.05) more Cd content in the roots and shoots (42% and 58%, respectively) compared to the control plants. We observed in the biomass study that the fresh and dry biomass of the So23-inoculated plant was lower than the noninoculated control plant. The increased Cd concentration in the plants might be a cause behind the lower biomass of So23-inoculated plants under Cd stress. Without Cd stress, the biomass shows no difference with control plants. However, Bc09-, E02-, Oj24- and mixed-inoculated plants had 53%, 21%, 54%, and 43% lower Cd contents, respectively, compared to the noninoculated Cd-control plants. The roots contained more Cd than the shoots (Figure 2A–C).

The TF of plants is the measurement of the ability to transfer metals from the roots to shoots [35]. This translocation depends largely on the types of metals and substrates [36]. The Bc09-inoculated plants had significantly (*p* < 0.05) higher TF (13%), whereas the mixed-inoculated plants had significantly (*p* < 0.05) (54%) lower TFs compared to the noninoculated Cd-control plants (Figure 2D). It is known that if the Cd concentration of at least 100 mg Kg^−1^ is accumulated [37] and the TF is greater than 1, then a plant is considered as hyper-accumulator [38]. In our study, the TF was less than 1 in both PGPB-inoculated and noninoculated Cd-control plants. Furthermore, the shoots had a Cd concentration of less than 100 mg Kg^−1^, while the roots contained more than 100 mg Kg^−1^ Cd. Therefore, this plant could be introduced as a Cd-accumulator rather than a Cd-hyperaccumulator.

### 2.3. Concentrations of IAA in Plants Determined through HPLC

**Figure 3:**
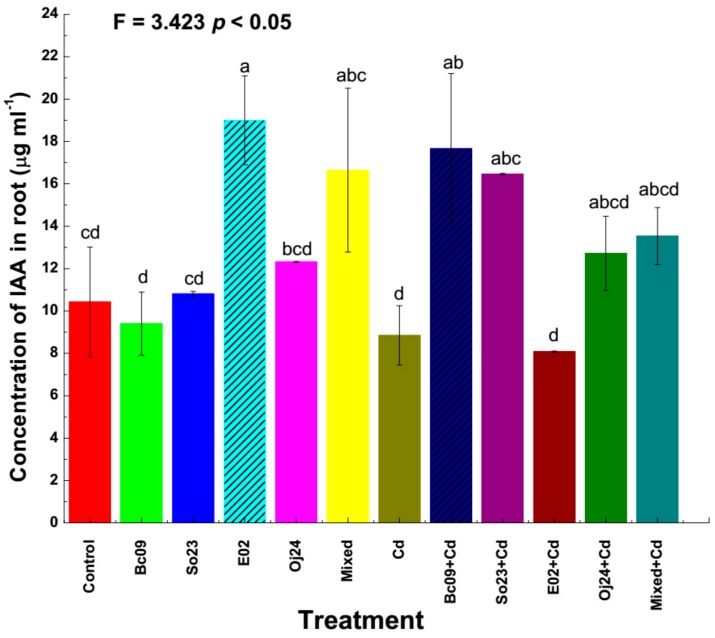
The IAA concentration in the root of switchgrass cv Alamo. Control = noninoculated control plant without Cd stress; Cd = noninoculated Cd-control plant with 20 µ mol L^−1^ CdCl_2_ stress; Bc09, So23, E02, Oj24, and Mixed (combination of the four strain) = plant inoculated with endophytic PGPB without Cd stress; and Bc09 + Cd, So23 + Cd, E02 + Cd, Oj24 + Cd, and Mixed + Cd (combination of the four strain) = plant inoculated with endophytic PGPB with 20 µ mol L^−1^ CdCl_2_ stress. The bar represents ±SE (n = 5). Columns not sharing a significance letter are significantly different according to Duncan’s Multiple Range Test at *p* < 0.05. The dash pattern indicates the biomass of that treatment is significantly different among the treatments.

The concentration of IAA was determined through high-performance liquid chromatography (HPLC), where the concentration of IAA was detected in the roots but not in the shoots in our triplicate experiments. The IAA concentration was higher in the PGPB-inoculated plants without Cd-amendment, with a difference ranging from 3.6% to 82.2%, except for the Bc09-inoculated plants. Under Cd-amended conditions, the noninoculated Cd-control and E02-inoculated plants contained lower IAA concentrations compared to the noninoculated control plants. The Bc09-, So23-, Oj24-, and mixed-inoculated plants contained 69.4%, 57.8%, 22%, and 29.8% higher IAA concentrations compared to those of the control plants, respectively (Figure 3). Among all the treatments, the E02-inoculated plants grown without Cd-exposure and Bc09-inoculated plants grown with Cd-exposure contained significantly (*p* < 0.05) higher IAA concentrations than the noninoculated control plants. Other treatments showed no significant differences among them.

### 2.4. Expression of the HSP70 Gene in PGPB-Inoculated and Noninoculated Switchgrass

**Figure 4:**
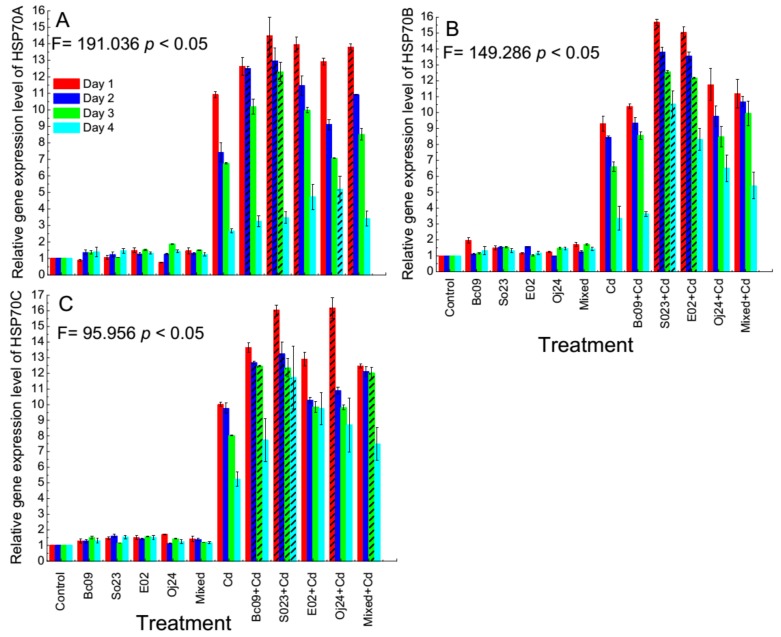
Expression of *HSP70* gene in the switchgrass cv Alamo. Relative gene expression level of (**A**) *HSP70*A = Pavir.5KG619900.1; (**B**) *HSP70*B = Pavir.9KG488300.1; (**C**) *HSP70*C = Pavir.5KG300400.1. Control = noninoculated control plant without Cd stress; Cd = noninoculated Cd-control plant with 20 µ mol L^−1^ CdCl_2_ stress; Bc09, So23, E02, Oj24, and Mixed (combination of the four strain) = plant inoculated with endophytic PGPB without Cd stress; and Bc09 + Cd, So23 + Cd, E02 + Cd, Oj24 + Cd, and Mixed + Cd (combination of the four strain) = plant inoculated with endophytic PGPB with 20 µ mol L^−1^ CdCl_2_ stress. The dash pattern indicates the biomass of that treatment is significantly different among the treatments. The expression of *HSP70* genes was determined by quantitative real-time PCR. FTSH protease 4 (FTSH4) was used as internal control. Each column represents the relative gene expression levels (mean value ± SD, with three biological repeats each with 3 technical repeats) calculated using 2^-∆∆CT^ method.

The relative gene expression level of three genes of the *HSP70* gene family, that is, Pavir.5KG619900.1 (*HSP70A*), Pavir.9KG488300.1 (*HSP70B*), and Pavir.5KG300400.1 (*HSP70C*), were analyzed by the qRT-PCR method and found to express in all of the plants after four consecutive days with and without Cd stress (Figure 4A–C). It should be noted that both with and without Cd stress, *HSP70* gene expression in the PGPB-inoculated plants was upregulated compared with that in the control plant. On the first day of Cd exposure, the *HSP70A* gene expression level was 10.9–14.5 fold higher in the noninoculated Cd-control and PGPB-inoculated plants compared to the noninoculated control plants. This gradually decreased after the second, third, and fourth day of exposure. In the second day, the gene expression level was 7.4–13 fold higher compared to the noninoculated control plants, which became 6.8–12.3 fold higher on the third day for the same treatment groups. Finally, on the fourth day the gene expression level was 2.7–5.2 fold higher compared to the noninoculated control plants (Figure 4A). Plants inoculated with So23 (on the first to third day), Bc09 (on the second day), E02 (on the first day), Oj24 (on the fourth day), and mixed (on the first day) had significantly (*p* < 0.05) higher levels of gene expression compared to the Cd-control.

In the case of *HSP70*B gene, on the first day of Cd exposure, the expression level was 9.3–15.7 fold higher in noninoculated Cd-control, PGPB-inoculated plants compared to the noninoculated control plants. The gene expression level was also observed to gradually decreased after second, third and fourth day of exposure to Cd. The expression was 8.4–13.8 fold higher; 6.6–12.6 fold higher; and 3.4–10.5 fold higher in noninoculated Cd-control and PGPB-inoculated plants compared to the noninoculated control plants (Figure 4B). The *HSP70*B gene expressed significantly more (*p* < 0.05) in the So23-inoculated plants on all four days. In addition, the gene also expressed significantly more (*p* < 0.05) in the E02-inoculated plant on the first three consecutive days, compared to the Cd-control.

In the case of the *HSP70*C gene, the expression level was 10–16.2 fold higher, 9.8–13.2 fold higher, 8–12.5 fold higher, and 5.2–11.7 fold higher in the noninoculated Cd-control and PGPB-inoculated plants after one day, two days, three days, and four days of Cd-exposure, respectively (Figure 4C), where a gradual decrease in gene expression was also observed in the last two days of Cd exposure compared to the first two days of exposure. The *HSP70*C gene expressed significantly more (*p* < 0.05) in the So23-inoculated plant on all the four consecutive days, in the Bc09-inoculated plant on the second and third day, in E02-inoculated plant on the first day, and in the mixed-inoculated plants on the fourth day.

### 2.5. Expression of HMA3 Gene in PGPB-Inoculated and Noninoculated Switchgrass

**Figure 5:**
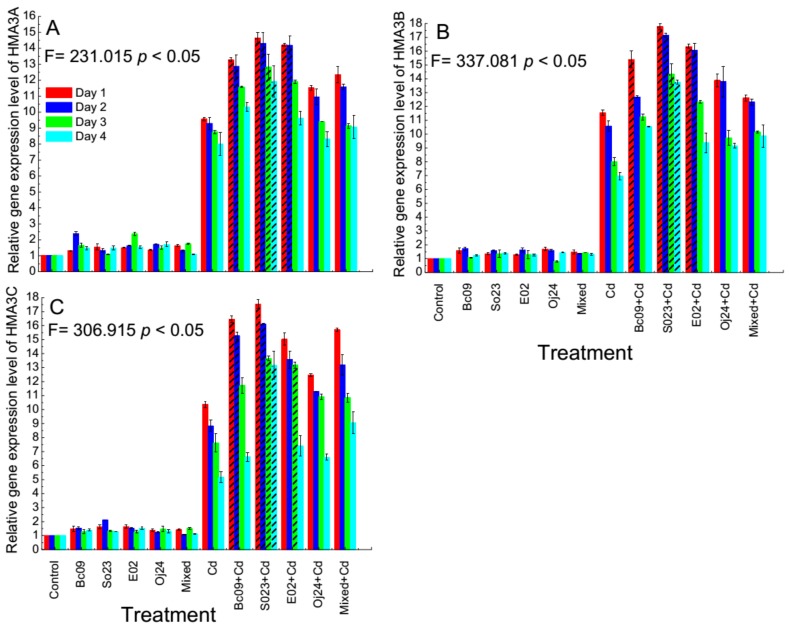
Expression of *HMA3* genes in the roots and shoots of the switchgrass cv Alamo. Relative gene expression level of (**A**) *HMA3*A = AP13CTG05330TIGR01512; (**B**) *HMA3*B = AP13CTG10982TIGR01512; (**C**) *HMA3*C = Pavir.Ba03387.1. Control = noninoculated control plant without Cd stress; Cd = noninoculated Cd-control plant with 20 µ mol L^−1^ CdCl_2_ stress; Bc09, So23, E02, Oj24, and Mixed (combination of the four strain) = plant inoculated with endophytic PGPB without Cd stress; and Bc09 + Cd, So23 + Cd, E02 + Cd, Oj24 + Cd, and Mixed + Cd (combination of the four strain) = plant inoculated with endophytic PGPB with 20 µ mol L^−1^ CdCl_2_ stress. The dash pattern indicates the biomass of that treatment is significantly different among the treatments. The expression of *HMA3* genes was determined by quantitative real-time PCR. FTSH protease 4 (FTSH4) was used as internal control. Each column represents the relative gene expression levels (mean value ± SD, with three biological repeats each with 3 technical repeats) calculated using 2^-∆∆CT^ method.

The relative gene expression level of three genes of the *HMA3*, that is, AP13CTG05330TIGR01512 (*HMA3A*), AP13CTG10982TIGR01512 (*HMA3B*), and Pavir.Ba03387.1 (*HMA3C*), was analyzed by the qRT-PCR method. Gene expressions were evaluated during four consecutive days after Cd exposure and were found to express in the PGPB-inoculated plants under both Cd-amended and Cd-nonamended conditions (Figure 5A–C).

In the case of the *HMA3*A gene, on the first, second, third and fourth day of exposure to Cd stress, the expression level was 9.6–14.6 fold higher, 9.3–14.3 fold higher, 8.8–12.8 fold higher, and 8–11.9 fold higher in the noninoculated Cd-control and PGPB-inoculated plants compared to the noninoculated-control plants (Figure 5A). The gene expressed significantly more in the So23-inoculated plant on all four consecutive days. Moreover, plants inoculated with Bc09 and E02 strain showed significant (*p* < 0.05) higher *HMA3*A gene expression on the first two days compared to the noninoculated Cd-control plant.

In case of *HMA3*B gene, the expression level was 11.5–17.8 fold higher, 10.6–17.1 fold higher, 8–14.3 fold higher, and 7–13.8 fold higher in noninoculated Cd-control and PGPB-inoculated plants compared to the noninoculated control plants on the first day, second day, third day, and fourth day of exposure to Cd stress (Figure 5B). After Cd exposure on all four consecutive days, the So23-inoculated plant showed significantly (*p* < 0.05) higher gene expression among all the treatments. Furthermore, E02-inoculated plant showed significantly (*p* < 0.05) higher gene expression on the first two days, whereas the Bc09-inoculated plants showed significantly (*p* < 0.05) higher gene expression on the first days among the four days of Cd-exposure.

In the case of the *HMA3*C gene, the expression level was 10.4–17.5 fold, 8.8–16.1 fold, 7.6–13.7 fold, and 5.2–13.2 fold higher in the noninoculated Cd-control and PGPB-inoculated plants after one day, two days, three days, and four days of Cd exposure (Figure 5C). The So23-inoculated plant showed significantly (*p* < 0.05) higher gene expression among all the treatments on all four consecutive days. Furthermore, Bc09-inoculated plant showed significantly (*p* < 0.05) higher gene expression on the first two days and the E02-inoculated plants showed significantly (*p* < 0.05) higher gene expression on the third day.

It should be noted that the gene expression level in the PGPB-inoculated plant was higher on the first day of Cd exposure and gradually decreased on the second, third and fourth day of exposure in case of all three *HMA3* genes.

### 2.6. PCA to Evaluate the Correlation between Plant Growth Parameters, Cd Concentrations, TF, IAA Concentrations, and HSP70 and HMA3 Gene Expression Level

**Figure 6:**
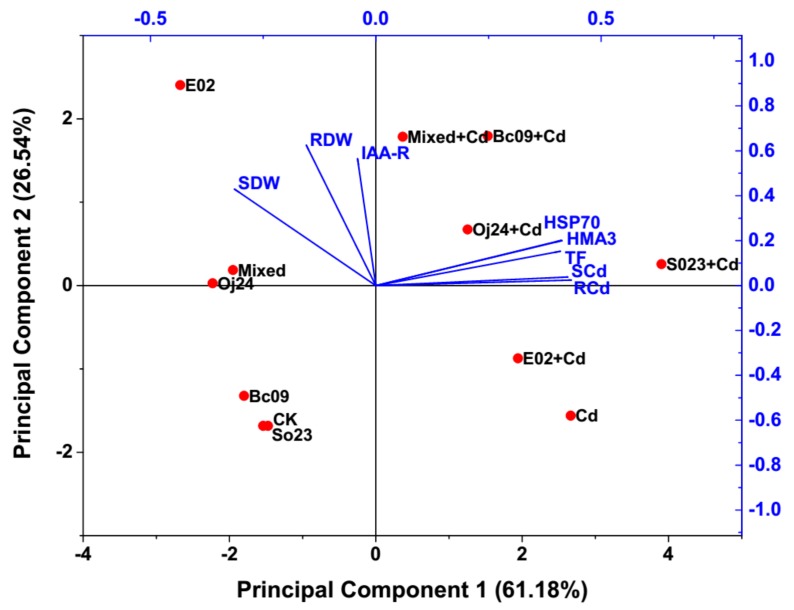
Principal Component Analysis (PCA) biplot based on correlation matrix. CK = noninoculated control with no Cd stress; Bc09, So23, E02, Oj24 and Mixed = PGPB (Bc09, So23, E02, Oj24 and combination of four)-inoculated Alamo with no Cd stress. Cd = noninoculated Cd-control under 20 µ mol L^−1^ CdCl_2_ stress; Bc09 + Cd, So23 + Cd, E02 + Cd, Oj24 + Cd and Mixed + Cd = PGPB (Bc09, So23, E02, Oj24 and combination of four)-inoculated Alamo under 20 µ mol L^−1^ CdCl_2_ stress. RDW = root dry weight, SDW = shoot dry weight, RCd = Cd concentration in root, SCd = Cd concentration in shoot, TF = translocation factor, IAA-R = Concentration of IAA in root, HSP70 = HSP70 gene expression level, HMA3 = HMA3 gene expression level.

There is more than one response variable in most research studies and variables need to be systematically analyzed to take advantage of the information about the relationships between them. PCA is used to simplify a number of related variables simultaneously. In the combined data set, PCA provided two principal factors, F1 (X-axis) and F2 (Y-axis), where the eigenvalue was > 1 and able to explain approximately 87.72% of the variability of the total data (PC 1 variance of 61.18% and PC 2 variance of 26.54%) (Figure 6). Figure 6 represents a biplot analysis of data into PCs where the concentration of Cd in the roots (RCd), the concentration of Cd in the shoots (SCd), TF, expression level of *HSP70* gene and *HMA3* in the plants corresponded to PC 1 and root dry weight (RDW), shoot dry weight (SDW), and concentration of IAA in the roots (IAA-R) corresponded to PC 2. The correlation matrix among these parameters is shown in Table 1. From Figure 6 and Table 1, it was observed that RDW and SDW were significantly positively correlated. IAA-R and RDW were also significantly positively correlated. SDW showed a significant negative correlation with RCd and SCd. However, RCd and SCd were significantly positively correlated. In addition, TF was also significantly positively correlated with RCd and SCd. Again, TF was significantly positively correlated with *HSP70* and *HMA3*. However, *HSP70* and *HMA3* exhibited highly significant positive correlations between them. Furthermore, *HSP70* and *HMA3* possessed a significant positive correlation with RCd and SCd. Guo et al. [39] also reported a significant positive correlation between *FIHMA3* (a gene homologous to *OsHMA3* in rice) gene expression and Cd accumulation in the roots of *Festulolium loliaceum* (Huds.), which might be responsible for reducing Cd toxicity through vacuolar sequestration of Cd into roots. From the PCA biplot (Figure 6), it was confirmed that the PGPB-inoculated plants and the noninoculated Cd-control plants under Cd stress were far from the same group of plants without Cd stress. These findings provide evidence that both *HSP70* and *HMA3* gene expression in the PGPB-inoculated plants, together with the beneficial activities of PGPB, such as the production of IAA, ACCD activities, and phosphate solubilization, etc. [27], under Cd stress played an important role in limiting the entry of or detoxifying the Cd in the cytoplasm to reduce the Cd toxicity.

## 3. Discussion

Heavy metals, for example, Cd, impose an imbalance in the cellular homeostasis by silencing necessary enzymes and hindering the functions of proteins [8]. In order to balance the homeostasis, plants respond to abiotic stressors by activating the stress response genes. The *HSP*s gene is one of the well-studied genes that is activated during intracellular stress conditions and maintained cellular homeostasis [16,40,41,42]. This finding has a good agreement with our current study, where the *HSP70* gene was expressed both under Cd stress and without Cd stress in our experimental results. The reason behind this interesting phenomenon might be in response to the *HSP*s gene in various biological systems, such as stabilization, appropriate folding, and assembly of the proteins under both favorable and unfavorable environmental conditions [6,43]. Notably, the *HSP70* gene was highly expressed under Cd stress, especially in the PGPB-inoculated plants, compared to the noninoculated plants. In addition, on the first day and second day, *HSP70* gene expression increased considerably after Cd stress in PGPB-inoculated and noninoculated Cd-control plants, and then its expression gradually reduced on the third and fourth days relative to the first two days. The reason may be the plant responded rapidly after exposure to Cd and, thus, may have increased its gene expression. Wang et al. [44] investigated *HSP70* gene expression in creeping bentgrass under prolonged heat stress and observed the presence of *HSP70* in all treatments before and after heat stress, although the expression was higher after stress. Recently, one of our research groups discovered that 22 *HSP* genes were highly expressed under Cd stress, and in *Arabidopsis,* one *HSP*-encoding gene was overexpressed and provided plants with Cd tolerance. These findings support the evidence that the *HSP* network re-establishes normal protein function and cellular homeostasis in switchgrass under Cd stress [16]. Several articles have described the adverse effects of high or low levels of Cd toxicity on plants by changing the protein profiles [45,46,47]. Under Cd stress, *HSP70* was differentially expressed in tomato roots [48] and young poplar leaves [49], with significantly increased expression in germinating rice seedlings [50] and *Arabidopsis thaliana* [51]. Interestingly, in our study, the gene expression level was significantly higher in the PGPB-inoculated plants under Cd stress. Therefore, our finding demonstrated evidence that PGPB might have a protective capability to combat against Cd stress. However, few reports exist in the case of *HSP70* gene expression in plant–microbe interactions, especially endophytic PGPB interactions, under Cd toxic conditions, whereas several reports have described that the overexpression of *HSP70* has a positive correlation with the acquisition of tolerance under heavy metal stress [8]. To our knowledge, this report is the first attempt to discover *HSP70* gene expression in the endophytic PGPB-inoculated switchgrass under Cd stress.

PGPB can enhance plant tolerance under abiotic stresses through a mechanism known as induced systemic tolerance (IST) [41,52], in which PGPB have several notable activities, including the synthesis of phytohormones, such as IAA [53], ACCD activities that allow the degradation of ethylene precursor to relieve plant stress, solubilization of insoluble phosphate and *HSP* gene expression [54]. In our study, we also observed similar results, as the production of IAA and *HSP70* gene expression increased in the plants under Cd stress compared to the control with no stress and, notably, the PGPB-inoculated plants under Cd stress had better results than the noninoculated Cd-control plants.

There is a substantial difference between switchgrass cv Alamo and rice. Alamo has genome sizes of 1600 Mb, is tetraploid, is an inbreeder and is a perennial. The genome size of rice is 373 Mb, and rice is a diploid, has an outcrossing breeding system and is an annual. Although genetic organization and habit are dissimilar, they possess significant synteny and collinearity [55,56]. In this study, we evaluated the expression level of the three rice homologous gene *HMA3* in switchgrass cv Alamo. To the best of our knowledge, this is the first time the expression of *HMA3* gene expression in switchgrass has been examined. We observed an upregulated gene expression level under Cd stress, which was similar to the *HMA3* gene expression results in rice [18]. It is important to mention that this gene was expressed both under Cd-amended and Cd-nonamended conditions in the switchgrass, while under Cd stress the expression was enhanced, especially in the PGPB-inoculated switchgrass. In addition, in the Cd-control and PGPB-inoculated plants, an enhanced level of *HMA3* gene expression was also noted on the first two days of Cd-exposure compared to the last two days of Cd-exposure, similar to *HSP70* genes. The mineral analysis of the Cd group showed that the Cd concentration were significantly lower in PGPB-inoculated Alamo compared to the noninoculated Cd-control plants. However, the exception was that the So23-inoculated plant showed significantly higher *HSP70* and *HMA3* gene expression but displayed higher Cd concentrations both in roots and shoots compared to the noninoculated Cd-control plants with less biomass achievement. In our previous study, we reported that So23 required a longer time than the other three bacterial strains to increase its growth rate under highly Cd-toxic conditions. Furthermore, uptake of Cd from high Cd containing sources was higher in the So23 strain [27]. Thus, we conclude that this characteristic of this bacterium might be a reason for the higher Cd content in plants. The *OsHMA2* promoter could control *OsHMA3* gene expression and, thus, reduce Cd accumulation in rice grains by sequestering more Cd into the vacuoles of different tissues after uptake by the root cells [3,17]. As a result of the enhanced sequestration of Cd in the vacuoles in the root cells, the Cd concentration decreased in the shoots. If the tonoplast localized transporter for Cd *OsHMA3* became functionless, then it would cause a reduced accumulation of Cd in the roots but a higher accumulation in the shoots [19]. However, functional *OsHMA3* induced vacuolar sequestration of Cd in the roots and accumulated more Cd, resulting in enhanced tolerances to Cd. In natural conditions, without stress this gene is usually expressed at a lower level [18]. In the present study, the PGPB-inoculated switchgrass gained more biomass and had reduced Cd concentrations compared to the noninoculated-Cd control. One of the underlying mechanisms behind this might be the expression of the *HMA3* gene. Extensive research is needed on this topic. Similar results were observed in the case of *AtHMA3* in *Arabidopsism*, a homolog of *OsHMA3*, which showed transport activity for Pb and Cd when expressed in a yeast mutant [57]. The underlying mechanism behind this transport-substrate affinity in switchgrass species needs to be investigated in the future. Previously, we reported bacterial plant growth-promoting activities, such as phytohormone IAA production, ACCD activities, phosphate solubilization, Cd tolerances and toxicity tests [27]. Currently, we were interested in observing the IAA production by PGPB-inoculated and noninoculated plants with and without Cd-amended conditions. Interestingly, we detected IAA in roots but not in the shoots. The IAA production was higher in the PGPB-inoculated plants than in the Cd-control plants under Cd stress.

In the PCA biplot and correlation matrix, we observed that the growth, Cd concentration in plant tissues, TF, IAA production, *HSP70,* and *HMA3* gene expression were correlated with each other. In addition, we observed differences in gene expression patterns between plants that were grown normally and under Cd stress; interestingly, the expression was higher in the PGPB-inoculated plants under Cd stress. From previous reports, it is known that a higher TF indicates increased Cd transportation in the shoots and roots. However, a lower TF is associated with reduced Cd accumulation in the shoots and roots [38]. This was reflected in the correlation analysis, where a significant positive correlation between TF, RCd, and SCd was observed. The gene expression of *HSP70* and *HMA3* in the plants were also positively correlated with TF. Finally, the results of this study demonstrated enhanced plant growth and biomass under Cd stress conditions, especially in the PGPB-inoculated plants compared to the noninoculated plants in this experiment. The PCA biplot also revealed that the PGPB-inoculated plants and noninoculated-control plants under Cd stress were far from the same group of plants without Cd stress. Based on their gene expression levels, *HSP70* and *HMA3* were more highly expressed in the single PGPB-inoculated plants than in the co-inoculated (mixed) plants under Cd stress (Figure 4 and Figure 5). The same and/or different PGPB in independently and/or in groups can trigger beneficial mechanisms differently under the same environmental situation and gene expression level, and proteomic profiles could also be modified in different manners [21]. However, the plant-PGPB interaction is dependent on plant genotype and bacterial strain [58]. Regardless of the mechanisms used, PGPB helps plants to enhance growth and yields; therefore, we obtained higher biomass in all the PGPB-inoculated plants compared to the Cd control plants under Cd stress.

This study concludes that PGPB has a profound influence on the expression of *HSP70* and *HMA3* gene in switchgrass under Cd stress. They are environmentally friendly agents to help plants during Cd toxic conditions and could assist in increase biomass production of switchgrass. Moreover, this will increase the success of unfavorable land use and intensify agricultural production.

## 4. Materials and Methods

### 4.1. Plant Cultivation, Harvest, Measurement of Biomass and Determination Cd Concentrations

Plant cultivation and harvest and the determination of Cd concentrations in plant tissues and TFs were carried out according to Begum et al. [27]. The PGPB strains were Bc09, So23, E02, Oj24, and a mixed culture of these four strains. The Cd stress that with the application of 20 µ mol L^−1^ CdCl_2_ for 4 days. The plants were grown in hydroponic culture in a greenhouse for 30 days from germination to harvest. There were two groups of plants: one group was under Cd stress conditions, and another group was grown without Cd stress. Each plant treatment had 6 replicate pots with 7 plants in each pot. A sampling of plants for the assessment of gene expression analysis was performed on the first day (instantly after exposure to Cd), the second day, the third day, and fourth day of exposure to Cd.

The concentrations of Cd and TFs inside plant tissues were calculated as follows [27]:

(1) $$\mathrm{Cd}\;\mathrm{concentration}\;\mathrm{in}\;\mathrm{plant}\;\mathrm{tissues}=\frac{\mathrm{Cd}\;\mathrm{root}\;\mathrm{or}\;\mathrm{shoot}\;\times \mathrm{Volume}}{\mathrm{Dry}\;\mathrm{weight}\;\mathrm{root}\;\mathrm{or}\;\mathrm{shoot}}$$

where “Cd root or shoot” is the inductively coupled plasma optical emission spectrometer (ICP-OES, Optima 2100 DV, Perkin Elmer, Gaithersburg, MD, USA) machine reading of the Cd concentration in the shoots or roots in mg L^−1^ and “Dry weight root or shoot” is the dry weight of roots or shoots in kg;

(2) $$\mathrm{TF}=\frac{\mathrm{Cd}\;\mathrm{shoot}}{\mathrm{Cd}\;\mathrm{root}}$$

where “Cd shoot” is the concentration of Cd in the shoots (mg kg ^−1^) and “Cd root” is the concentration of Cd in the roots (mg kg^−1^).

### 4.2. Determination of IAA through HPLC

IAA was determined in the plant samples through HPLC (Agilent Technologies, Waldbronn, Karlsruhe, Germany) performed following the method described by Cui et al. [59]. One gram of root and shoot samples were measured and immediately frozen in liquid nitrogen for rapid cooling to stop the metabolic activities and then stored at –80 °C. Then, the samples were ground in 5 mL of 50% precooled methanol. The ground samples were then incubated at 4 °C for 12 hr followed by centrifugation (Dynamic Dynamica Scientific Ltd., Suzhou, China) at 10,000 *g* for 10 min at 4 °C. The supernatant was transferred to a fresh tube and stored at 4 °C. To the sample, 3 mL of 50% precooled methanol was added and incubated at 4 °C for 12 hr. After centrifugation at 10,000 *g* for 10 min at 4 °C, the supernatant was collected and stored with the previously collected supernatant. To absorb the phenolic substances and pigments 0.2 g polyvinyl-poly pyrrolidone (PVPP) was added to the extract and the extract was incubated in a shaker incubator (Crystal IS-RDV1, Dallas, USA) at 4 °C for 60 min. Again the samples were centrifuged at 10,000 *g* for 10 min at 4 °C. The supernatant was collected and then passed through a C18 column (Sep-Pak Cartridges, Waters Corporation, Milford, MA, USA). The samples were then freeze-dried with a continuous supply of N_2_ gas. Then, the lyophilized samples were dissolved by adding 2.5 mL 50% precooled methanol. The dissolved samples were then filtered through a 0.22 µm ultrafiltration membrane. An aliquot was then injected into the high-performance liquid chromatography (HPLC) instrument. The chromatographic conditions for the Agilent 1290 Infinity system (Agilent Technologies, Waldbronn, Karlsruhe, Germany) were as follows: The mobile phase was 0.6% acetic acid (volume fraction) and chromatographic grade methanol gradient elution; the column temperature was 35 °C; the injection volume was 2 µL; the flow rate was 0.3 mL min^−1^; the detection wavelength was 254 nm.

### 4.3. Gene Expression of HSP70 and HMA3 in PGPB-Inoculated and Noninoculated Switchgrass

#### 4.3.1. Total RNA Extraction

Plant tissue (1 g fresh seedlings) was ground into a fine powder using liquid nitrogen. Total RNA was isolated and purified separately according to the manufacturer’s protocol using the Spin Column Plant Total RNA Purification Kit (Sangon Biotech, Co. Ltd. Shanghai, China). Extracted RNA was quantified using a Nanodrop ND-2000 spectrophotometer (Nanodrop Technologies, Inc., Wilmington, DE, USA). The 260/280 nm ratio of samples ranged from 1.9 to 2.1, and the average RNA integrity number (RIN) was over 8. These results indicated that the extracted RNA was of high quality without any apparent degradation and was suitable for further cDNA synthesis.

#### 4.3.2. Reverse Transcriptional Polymerase Reaction (RT-PCR)

A HiScript^TM^ Q RT SuperMix for qPCR (Vazyme, Nanjing, China) was used to synthesize the cDNAs. The synthesized cDNA was diluted 20 times with distilled water for use in qRT-PCR.

#### 4.3.3. Quantitative Real-time PCR (qRT-PCR)

The qRT-PCR was applied to analyze the relative expression level of a candidate gene in diluted cDNA samples. The primer sequences for the *HSP70*, *HMA3,* and the internal control FTSH protease 4 (FTSH4) genes are given in Table 2. All reactions were conducted in three biological replicates, and the reactions without template were used as negative controls. A Roche-480 system (Roche, Rotkreuz, Switzerland) was used to perform qRT-PCR using a final volume of 20 μL reaction mixture. The qRT-PCR program began with a denaturation step first (95 °C, 1 min), and then continued with 40 amplification cycles, which were programmed as 95 °C for 5 sec, 57 °C for 30 sec, and 72 °C for 30 sec. The CT values for the target and standard control genes were retrieved from the qRT-PCR system, and the comparative threshold 2^-∆∆CT^ method was applied to quantify the relative expression of the given gene [60,61].

### 4.4. PCA to Evaluate the Correlation between Plant Growth Parameters, Cd Concentrations, TFs, IAA Concentration, and HSP70 and HMA3 Gene Expression Levels

A PCA was performed to determine the correlation between plant growth parameters, Cd concentrations, TFs, IAA concentration, and *HSP70* and *HMA3* gene expression levels. The original variables in a linear combination are called the principal component to illustrate the variation in a particular orthogonal dimension. To determine the correlation of each principal component with each of the original variables, the Pearson bivariate correlation was performed [62].

### 4.5. Statistical Analyses

All data were statistically analyzed using IBM SPSS software version 23 (SPSS Inc.). The data are presented as the mean values with standard errors. The analysis was performed using the Duncan Multiple Range Test (DMRT). Significant differences were analyzed by one-way ANOVA at *p* < 0.05. The correlation matrix was constructed using Pearson bivariate correlation analysis using IBM SPSS software version 23 (SPSS Inc.). Graph preparation and principal component analysis (PCA) were performed using OriginPro 9.0 software (Origin Lab, Corporation, Northampton, USA).

## 5. Conclusions

Application of beneficial endophytic PGPB in agriculture seems to be a good approach in the future to ensure the high yield potential of plants and food safety. To the best of our knowledge, this is the first report on *HSP70* and *HMA3* gene expression in the PGPB-inoculated switchgrass under Cd stress. These results will provide information for future research on heavy metal resistance in plants with the assistance of PGPB.

## Figures and Tables

**Table 1:** Correlation matrix among growth parameters, concentrations of Cd, TF, concentrations of IAA, *HSP70,* and *HMA3* gene expression in switchgrass. RDW = root dry weight, SDW = shoot dry weight, RCd = Cd concentration in root, SCd = Cd concentration in shoot, TF = translocation factor, IAA-R = Concentration of IAA in root, *HSP70* = *HSP70* gene expression level, *HMA3* = *HMA3* gene expression level. The correlation matrix was constructed using Pearson bivariate correlation analysis.

Parameter	RDW	SDW	RCd	SCd	TF	IAA-R	*HSP70*	*HMA3*
RDW	1							
SDW	0.830 **	1						
RCd	–0.293	−0.627 *	1					
SCd	–0.304	−0.655 *	0.970 **	1				
TF	–0.109	–0.495	0.797 **	0.821 **	1			
IAA-R	0.685 *	0.417	–0.039	0.083	0.083	1		
*HSP70*	–0.013	–0.379	0.857 **	0.788 **	0.888 **	0.02	1	
*HMA3*	–0.017	–0.404	0.858 **	0.796 **	0.883 **	0.043	0.993 **	1

* Correlation is significant at the 0.05 level (2-tailed). ** Correlation is significant at the 0.01 level (2-tailed).

**Table 2:** The primer sequences.

Gene Name	Gene ID	Primer Sequences (5′ to 3′)
HSP70	Pavir.5KG619900.1 (*HSP70A*)	GAGCTGTGCAAGAGCATCAA TTCTTGGTTGGGATGGTGGT
	Pavir.9KG488300.1 (*HSP70B*)	ATCGACTTCTACGCGACCAT CTGCGACTTGTCCATCTTGG
	Pavir.5KG300400.1 (*HSP70C*)	AAGATCACCATCACCAGCGA CGTACGTCTCCAGCTTGTTG
HMA3	AP13CTG05330TIGR01512 (*HMA3A*)	GTGACCAAGTCATGGGAGGA GTGCAACAGCCAAAGAAAGC
	AP13CTG10982TIGR01512 (*HMA3B*)	GGAAGACTGCACGAACCATC CACAGCCCTTGTTGCTAGTC
	Pavir.Ba03387.1 (*HMA3C*)	GTTCTGGGAGCACAGGACAT AGTTCCCGTCTTGTCGAATG
FTSH4 (Internal control)		TGGATGGCTTTAAGCAGAATGA CAAAACGCCCAGGTCTGACT
